# The novel hypoxia-inducible factor-1*α* inhibitor IDF-11774 regulates cancer metabolism, thereby suppressing tumor growth

**DOI:** 10.1038/cddis.2017.235

**Published:** 2017-06-01

**Authors:** Hyun Seung Ban, Bo-Kyung Kim, Hongsub Lee, Hwan Mook Kim, Dipesh Harmalkar, Miso Nam, Song-Kyu Park, Kiho Lee, Joon-Tae Park, Inhyub Kim, Kyeong Lee, Geum-Sook Hwang, Misun Won

**Affiliations:** 1 Metabolic Regulation Research Center, KRIBB, Daejeon 305-806, Korea; 2 Biomolecular Science, University of Science and Technology, Daejeon 305-350, Korea; 3 Personalized Genomic Medicine Research Center, KRIBB, Daejeon 305-806, Korea; 4 Drug Discovery Team, ILDONG Pharmaceutical Co. Ltd, Hwaseong 445-811, Kyungi-do, Korea; 5 College of Pharmacy, Gachon University, Incheon 406-840, Korea; 6 College of Pharmacy, Dongguk University-Seoul, Goyang 410-820, Korea; 7 Integrated Metabolomics Research Group, Western Seoul Center, Korea Basic Science Institute, Seoul, Korea; 8 College of Pharmacy, Korea University, Sejong City 30019, Korea; 9 Functional Genomics, University of Science and Technology, Daejeon 305-350, Korea

## Abstract

HIF-1 is associated with poor prognoses and therapeutic resistance in cancer patients. We previously developed a novel hypoxia-inducible factor (HIF)-1 inhibitor, IDF-11774, a clinical candidate for cancer therapy. We also reported that IDF-1174 inhibited HSP70 chaperone activity and suppressed accumulation of HIF-1*α*. In this study, IDF-11774 inhibited the accumulation of HIF-1*α in vitro* and *in vivo* in colorectal carcinoma HCT116 cells under hypoxic conditions. Moreover, IDF-11774 treatment suppressed angiogenesis of cancer cells by reducing the expression of HIF-1 target genes, reduced glucose uptake, thereby sensitizing cells to growth under low glucose conditions, and decreased the extracellular acidification rate (ECAR) and oxygen consumption rate of cancer cells. Metabolic profiling of IDF-11774-treated cells revealed low levels of NAD^+^, NADP^+^, and lactate, as well as of intermediates in glycolysis and the tricarboxylic acid cycle. In addition, we observed elevated AMP and diminished ATP levels, resulting in a high AMP/ATP ratio. The level of AMP-activated protein kinase phosphorylation also increased, leading to inhibition of mTOR signaling in treated cells. *In vivo* xenograft assays demonstrated that IDF-11774 exhibited substantial anticancer efficacy in mouse models containing KRAS, PTEN, or VHL mutations, which often occur in malignant cancers. Collectively, our data indicate that IDF-11774 suppressed hypoxia-induced HIF-1*α* accumulation and repressed tumor growth by targeting energy production-related cancer metabolism.

Most cancer cells produce energy by glycolysis rather than mitochondrial oxidative phosphorylation, regardless of oxygen availability; this phenomenon is termed the Warburg effect.^1^ Specifically, this metabolic phenotype of cancer is regulated by the HIF-1, PI3K, p53, MYC, and AMP-activated protein kinase (AMPK)-liver kinase B1 pathways. Although HIF-1*α* is rapidly degraded under normoxic conditions, this protein is stabilized and dimerizes with the HIF-1*β* subunit in the nucleus under conditions of hypoxia.^2, 3^ These HIF-1*α*/*β* heterodimers subsequently bind to hypoxia-response elements (HREs) (5′-RCGTG-3′, where R is A or G) in the promoters of target genes involved in angiogenesis, metastasis, and resistance to apoptosis, thereby activating their transcription.^4^ In addition, HIF-1 activates glycolysis by facilitating the transcription of metabolic genes such as glucose transporters (GLUTs), hexokinase, pyruvate kinase M2, and lactate dehydrogenase A, leading to the reprograming of cancer cell metabolism.^4, 5^ Therefore, inhibition of HIF-1 could impair the metabolic adaptability of cancer cells and render them sensitive to cancer therapy.^6^

Although many efforts have been made to develop HIF-1 inhibitors, only few have reached clinical trials.^7, 8^ In particular, BAY 87-2243, a mitochondrial complex I inhibitor, was shown to reduce hypoxia-induced HIF-1*α* accumulation and suppress tumor growth in an H460 xenograft model;^9^ PX-478 was found to prevent hypoxia-mediated HIF-1 signaling by inhibiting HIF-1 translation,^10^ and to exert antitumor activity in various human cancer cell xenograft models;^11^ and KCN-1, a benzopyran analog, suppresses HIF-1 activity by disrupting the interaction of HIF-1*α* with the transcriptional coactivator p300 in glioma cells.^12^ In addition, NSC-134754 was found to reduce HIF-1 activity and tumor growth in a prostate cancer xenograft model.^13^

In previous studies, we reported the establishment of HIF-1 inhibitors based on the aryloxyacetylamino benzoic acid scaffold.^14, 15, 16^ From lead optimization studies, we recently developed an orally administered HIF-1 inhibitor, IDF-11774, which has been approved as a clinical candidate for a phase I study by the Korea Food and Drug Administration.^17^ Using chemical probes, we previously demonstrated that IDF-11774 inhibits HSP70 chaperone activity by binding to its allosteric pocket, rather than the ATP-binding site in its nucleotide-binding domain.^18^ The HSP70 family is reported to be associated with malignancy, clinical cancer stage, and poor prognosis of various cancers.^19, 20^

Here, we further analyze the anticancer efficacy of IDF-11774 *in vitro* and *in vivo*, as well as its mode of action. Our data demonstrate that IDF-11774 reduced cancer cell growth through the regulation of cancer glycolytic metabolism and energy production. IDF-11774 showed significant antitumor efficacy in xenograft assays of various cancer models bearing drug resistance mutations, suggesting that IDF-11774 may be used by itself or in combination with other agents in cancer therapeutics.

## Results

### IDF-11774 inhibits HIF-1*α* accumulation and suppresses angiogenesis

**Figure 1:**
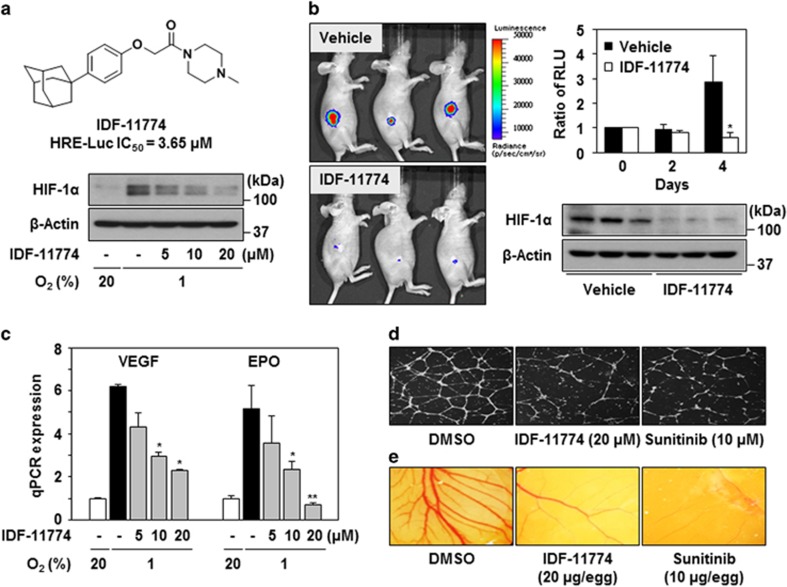
IDF-11774 inhibits HIF-1*α* accumulation in HCT116 cells. (**a**) Structure of IDF-11774 and its effect on HIF-1*α* accumulation, as determined by western blot analysis. (**b**) *In vivo* bioluminescence imaging of HIF-1 activity. The relative luminescence in live tumors and HIF-1*α* levels in tumor tissue were measured after treatment for 4 days. Data are presented as the means and standard deviations of the results from three independent experiments; *P<0.05, compared with vehicle control. (**c**) Quantitative real-time PCR analysis of the mRNA expression levels of HIF-1*α* target genes in HCT116 cells treated with IDF-11774 for 18 h. *P<0.05 and **P<0.01, compared with the untreated hypoxia control. (**d**) *In vitro* tube formation: HUVECs were treated with DMSO, IDF-11774, or sunitinib under 1% O_2_ for 24 h. (**e**) CAM assay: *in situ* inhibition of angiogenesis in the chicken embryo by treatment with IDF-11774 (20 *μ*g per egg) or sunitinib (10 *μ*g per egg)

Previously, we screened a focused library of aryloxyacetylamino benzoic acid scaffolds and performed lead optimization for the identification of an HIF-1*α* inhibitor. We found that IDF-11774 reduced the HRE-luciferase activity of HIF-1α (IC_50_=3.65 *μ*M) and blocked HIF-1*α* accumulation under hypoxic conditions in HCT116 human colon cancer cells (Figure 1a and Supplementary Figure S1a). We next evaluated the effect of IDF-11774 on HIF-1α accumulation *in vivo* using a bioluminescence imaging assay. Luciferase activity and HIF-1α accumulation were strongly suppressed in the tumors of mice treated by oral administration of IDF-11774, compared with the control (Figure 1b and Supplementary Figure S1b).

Subsequently, we found that IDF-11774 inhibited the expression of the HIF-1 target genes *VEGF* and *EPO*, which are involved in angiogenesis, but not of the gene encoding HIF-1*α* itself (Figure 1c). Therefore, we investigated the effects of the inhibitor on angiogenesis using both *in vitro* and *in vivo* assays. In the *in vitro* tube formation assay, human umbilical vascular endothelial cells (HUVECs) treated with IDF-11774 showed reduced capillary network formation on Matrigel, similar to that observed with sunitinib, the positive control (Figure 1d). This IDF-11774-mediated inhibition of *in vitro* tube formation was rescued by the addition of VEGF (Supplementary Figure S1c). Similarly, chick embryo chicken chorioallantoic membrane (CAM) analyses revealed treatment with IDF-11774 and sunitinib resulted in reduced vessel formation within the CAM *in vivo*, compared with that observed in the negative control (Figure 1e). These results strongly indicate that IDF-11774 potently represses angiogenesis.

### IDF-11774 inhibits glucose-dependent cancer metabolism

**Figure 2:**
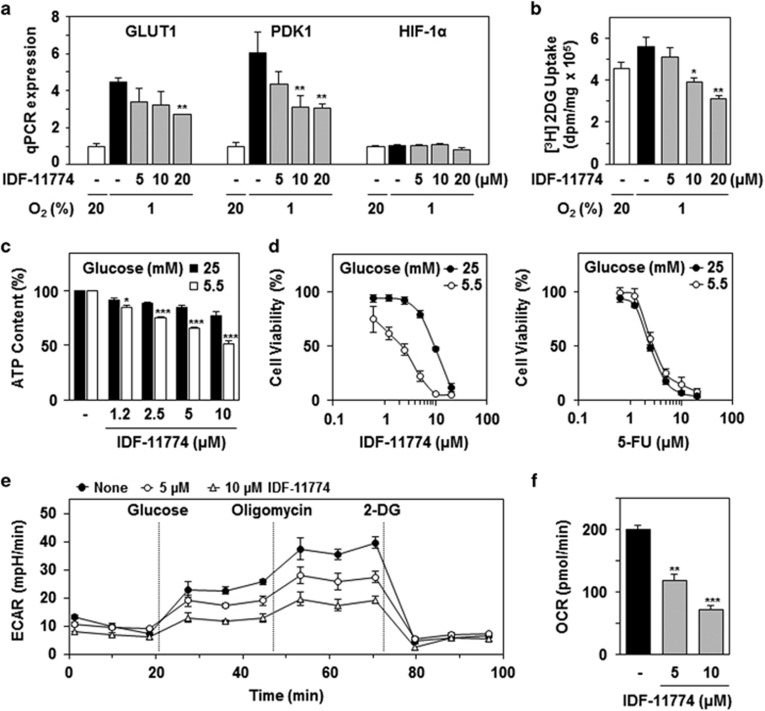
Effect of IDF-11774 on glycolytic energy metabolism and cell growth. (**a**) Effect of IDF-11774 on mRNA expression after treatment of HCT116 cells with IDF-11774 for 6 h; *P<0.05 and **P<0.01, compared with the untreated hypoxia control. RPL13A was used as a control. (**b**) Effect of IDF-11774 on glucose transport using 2DG. (**c**) Levels of glucose-dependent ATP production in HCT116 cells cultivated in the presence of IDF-11774 for 12 h. (**d**) Effect of IDF-11774 on glucose-dependent cell growth. The growth of cells treated with IDF-11774 or 5-FU for 72 h was compared in media containing 25 or 5.5 mM glucose. Data are presented as the means and standard deviations of the results from three independent experiments; *P<0.05, **P<0.01, and ***P<0.001, compared with 25 mM glucose. (**e** and **f**) Effects of IDF-11774 on (**e**) glycolysis and (**f**) mitochondrial respiration

We next revealed that IDF-11774 treatment resulted in reduced mRNA expression of GLUT1 and pyruvate dehydrogenase kinase 1 (PDK1), which are targets of HIF-1 (Figure 2a). Therefore, we assessed whether IDF-11774 affected the metabolism of HCT116 cells. Glucose uptake assay analysis demonstrated that IDF-11774 markedly suppressed the cellular uptake of [^3^H]2-deoxyglucose (2DG) (Figure 2b). In addition, intracellular ATP levels were significantly reduced in the presence of IDF-11774 and were affected to a greater degree under low glucose conditions (5.5 mM) (Figure 2c). Indeed, cells treated with IDF-11774 in low glucose medium exhibited stronger growth inhibition than cells treated in high glucose (25 mM) (Figure 2d). Conversely, 5-fluorouracil (5-FU) and sorafenib exerted no glucose-related inhibitory effects on the growth of cancer cells (Figure 2d and Supplementary Figure S2).

Subsequently, the effect of IDF-11774 on the ECAR, which is an indicator of glycolysis activity, was determined using an XF-24 Extracellular Flux Analyzer (Seahorse Biosciences, North Billerica, MA, USA). Treatment of cells with IDF-11774 inhibited both glucose-mediated basal and oligomycin-mediated ECAR in a dose-dependent manner (Figure 2e), suggesting that IDF-11774 inhibits glycolysis in HCT116 cells. We further investigated the effects of IDF-11774 on mitochondrial respiration by measuring the oxygen consumption rate (OCR). IDF-11774 significantly inhibited OCR in a concentration-dependent manner (Figure 2f), demonstrating that this compound also blocks mitochondrial respiration.

### Metabolic profiling of cells treated with IDF-11774

**Figure 3:**
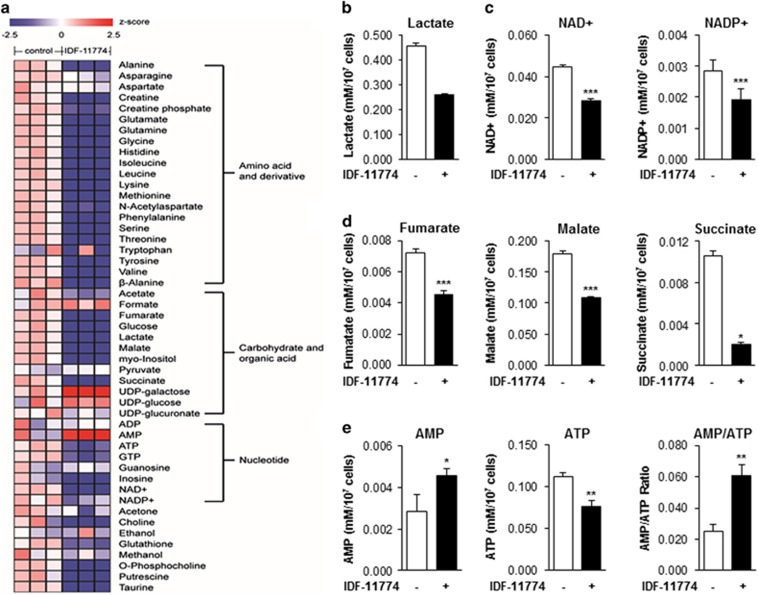
Metabolic profile of cells treated with IDF-11774 under hypoxic conditions. (**a**) Heatmaps of quantified metabolites in HCT116 cells treated with or without 10 *μ*M IDF-11774 for 12 h. (**b**–**e**) Levels of (**b**) lactate, (**c**) NAD^+^ and NADP^+^, (**d**) TCA cycle intermediate metabolites, and (**e**) AMP and ATP in HCT116 cells treated with or without 10 *μ*M IDF-11774 for 12 h. Data are presented as the means and standard deviations of the results from three independent experiments; *P<0.05; **P<0.01 and ***P<0.001, compared with the untreated control

To confirm that IDF-11774 affects the metabolism of HCT116 cells, we examined the metabolite profiles of cells treated with IDF-11774 under hypoxic condition via ^1^H-NMR spectroscopy. Treatment of cells with IDF-11774 for 12 h did not affect HCT116 cell growth (Supplementary Figure S3). A total of 49 metabolites, including amino acids, carbohydrates, organic acids, and nucleotides, in HCT116 cell extracts were identified and quantified under the test conditions (Figure 3a). Notably, IDF-11774 treatment resulted in reductions in the levels of many glycolysis and tricarboxylic acid (TCA) cycle metabolites. In particular, IDF-11774 significantly reduced the levels of intracellular lactate, an end product of glycolysis (Figure 3b), and NAD^+^ and NADP^+^, which are metabolites related to nicotinamide metabolism (Figure 3c). In addition, the levels of TCA cycle intermediates such as fumarate, malate, and succinate were apparently reduced in HCT116 cells treated with IDF-11774, compared with the control (Figure 3d). Furthermore, IDF-11774 treatment resulted in increased cellular AMP levels and decreased ATP levels, resulting in an elevated AMP/ATP ratio (Figure 3e). These data suggest that growth inhibition in HCT116 cells treated with IDF-11774 under hypoxic conditions was the result of inhibition of energy production metabolism.

**Figure 4:**
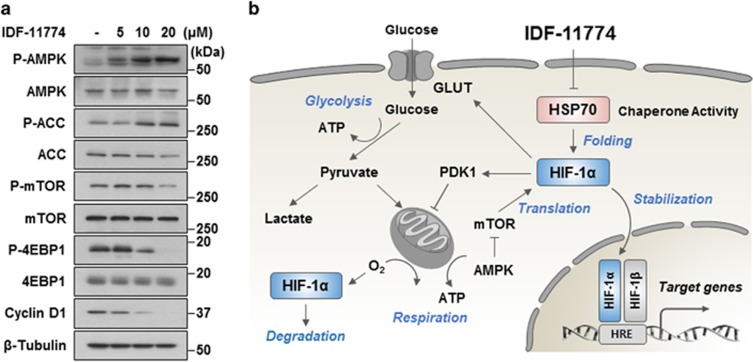
Effect of IDF-11774 on energy metabolism. (**a**) Western blot analysis of the expression levels of proteins involved in AMPK activation in the presence or absence of IDF-11774. (**b**) Model depicting the mode of action of IDF-11774: IDF-11774 suppresses HIF-1*α* accumulation, thereby inhibiting glucose-dependent energy metabolism and mitochondrial respiration in cancer cells. HIF-1*α* accumulation was reduced by the enhanced degradation of HIF-1*α* consequent to increased local oxygen concentrations and the translational attenuation of HIF-1*α* by mTOR inactivation

Finally, we performed western blot analysis to determine the degree of IDF-11774-mediated activation of AMPK signaling consequent to the elevation of the AMP/ATP ratio. IDF-11774 treatment resulted in increased phosphorylation of AMPK, which inactivates ACC and suppressed the phosphorylation of mTOR and 4EBP1, thereby ultimately attenuating the translation of HIF-1α (Figure 4a). These results suggest that IDF-11774 inhibits glucose-dependent cancer metabolism through positive feedback regulation of HIF-1α inhibition (Figure 4b).

### IDF-11774 suppresses tumor growth in various cancer xenograft models

**Figure 5:**
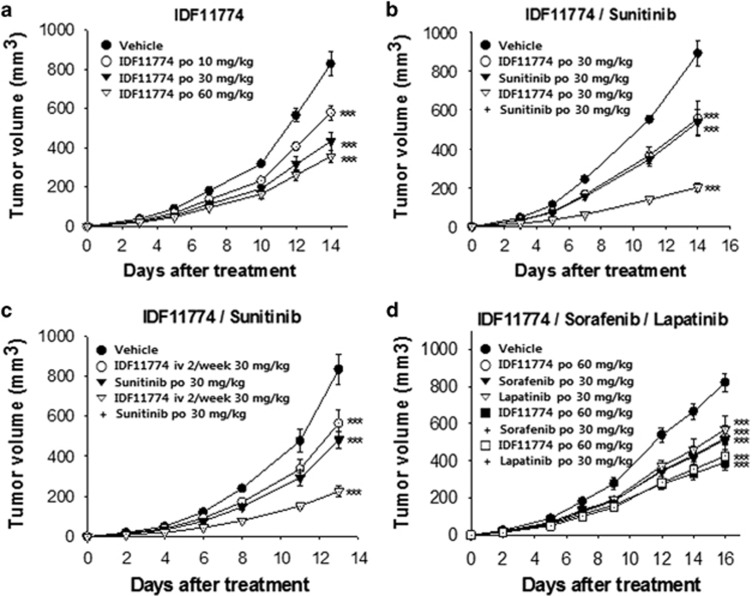
Antitumor efficacy of IDF-11774 in HCT116 xenograft models. Graphic depictions of tumor size in xenograft model mice in the presence or absence of (**a**) IDF-11774 (per oral (p.o.), 10, 30, or 60 mg/kg, once a day (q.d.)), (**b**) IDF-11774 and sunitinib (p.o., 30 mg/kg each, q.d.), (**c**) IDF-11774 (intranvenously (i.v.), 2/wk; 30 mg/kg, q.d.) and sunitinib (p.o., 30 mg/kg, q.d.), or (**d**) IDF-11774 (p.o., 60 mg/kg, q.d.) and sorafenib or lapatinib (p.o., 30 mg/kg each, q.d.). Data are presented as the means and standard deviations of the results from five independent experiments; ***P<0.001, compared with vehicle control

To evaluate the *in vivo* antitumor efficacy of IDF-11774, we performed a xenograft assay using HCT116 cells (Figures 5a–d and Supplementary Table S1). When IDF-11774 was orally administered daily for two weeks, significant dose-dependent tumor regression was observed in the mouse model (Figure 5a). We then tested whether IDF-11774 could be used for combination therapy with other agents to enhance its anticancer efficacy in an HCT116 xenograft model. The combination of IDF-11774 with the multikinase inhibitor sunitinib resulted in a significant increase in anticancer efficacy, compared with individual treatment with either agent (Figure 5b). When intravenous treatment of IDF-11774 (intravenously, twice a week) was combined with sunitinib (per oral, once a day), a similar enhancement of antitumor efficacy was obtained, compared with each treatment alone (Figure 5c). In addition, the combined treatment of IDF-11774 with the multikinase inhibitors sorafenib or lapatinib also showed enhancement in anticancer efficacy (Figure 5d). Notably, however, no significant weight loss or side effects, such as skin ulcers or other severe symptoms, were observed in any of the treated mice.

To investigate additional applications of IDF-11774, the effects of IDF-11774 on other cancer cells were investigated. IDF-11774 inhibited HIF-1α accumulation and the growth of various cancer cells (Supplementary Figures S4a and S4b). Furthermore, we investigated the *in vivo* antitumor efficacy of IDF-11774 in various xenograft models (Table 1 and Supplementary Figure S5). IDF-11774 exhibited strong efficacy in inhibiting the growth of A549 cell tumor, which contain a KRAS mutation. IDF-11774 also inhibited the tumor growth of NCI-H1975 cells bearing an EGFR T790M mutation and wild-type KRAS. Notably, IDF-11774 demonstrated good response in both PC-3 prostate cancer cells, which are PTEN-null, as well as MIA-PaCa-2 pancreatic cancer cells. Last, IDF-11774 significantly suppressed the tumor growth of Caki-1 renal cancer cells containing wild-type VHL and of 786-O cells with truncated VHL. Collectively, IDF-11774 showed significant antitumor efficacy in xenograft models of tumors harboring various mutations.

## Discussion

Cancer cells show alterations in cellular metabolism, such as aerobic glycolysis, high fatty acid synthesis, and rapid glutamine metabolism, which is linked to therapeutic resistance.^1, 21, 22^ Accordingly, mutations and alterations in several metabolism-related enzymes, including IDH1, IDH2, SDH, FH, and PKM2, have been discovered in various cancers.^23^ Therefore, it has been suggested that targeting of metabolic pathways likely represents an efficient strategy to improve antitumor efficacy and overcome therapeutic resistance in the development of anticancer drugs.^24^ In particular, metabolic inhibitors targeting GLUTs, PDK1, and glycolytic enzymes have been shown to enhance anticancer efficacy and mitigate drug resistance.^24^

We have developed an orally administered anticancer agent, IDF-11774, which reduced HIF-1*α* accumulation both *in vitro* and *in vivo*. IDF-11774 suppressed *in vitro* tube formation and vascularization in an *in vivo* CAM assay, indicating an inhibition of angiogenesis. In a previous report, IDF-11774 inhibited HSP70 chaperone activity, resulting in suppression of HIF-1α refolding.^25^ Moreover, IDF-11774 reduced the OCR and ATP production, thereby increasing intracellular oxygen tension to stimulate HIF-1α degradation. Furthermore, IDF-11774 suppressed GLUT1 expression and ECAR, resulting in sensitization of cells to growth under low glucose conditions, and significantly inhibited mitochondrial respiration, resulting in increased local oxygen tension to promote the proteasomal degradation of HIF-1*α*.^25^ It has been reported that HIF-1 inhibition increases mitochondria-mediated oxygen consumption due to reduction of PDK1 expression.^26^ Therefore, our observation of decrease in OCR in the presence of IDF-11774 is likely the effect of HSP70 inhibition, which is HIF-1*α* -independent.

In a cell culture system, short exposure to hypoxic conditions resulted in increased HIF-1*α* accumulation, while prolonged exposure to hypoxia resulted in reduced accumulation of this protein.^27^ In our study, to understand the effects of IDF-11774 on HIF-1*α* expression, we evaluated the levels of HIF-1*α* production in HCT116 cells after exposure to hypoxia for 6 h, at which peak levels of HIF-1*α* were previously observed. Indeed, Luo *et al.* suggest that HIF-1*α* is degraded by CHIP/HSP70-mediated proteasomal degradation in HEK293 cells during prolonged exposure to hypoxic conditions.^28^ It appears that coordinated positive and negative regulatory mechanisms control the transcription and degradation of HIF-1*α* to maintain cell homeostasis. Consistent with this conclusion, several reports have described distinct regulatory mechanisms underlying the degradation of HIF-1*α* in cancer cells.^29, 30, 31^ In addition, complex relationships between transcriptional activation of HSP70 family members by HIF-1*α* have been reported.^32, 33^

Next, to confirm the effect of IDF-11774 on cancer metabolism, we examined the metabolic profile of cells treated with IDF-11774. IDF-11774 significantly reduced the intracellular lactate level, indicating a decrease in glycolysis. In addition, there was a significant decrease in the levels of intermediates in glycolysis and the TCA cycle, as well as in amino acids, which are necessary for biosynthesis of the building blocks of cell growth. Furthermore, cellular concentrations of NAD^+^ and NADP^+^ decreased in the presence of IDF-11774, compared with the control. NAD^+^ is converted to NADH by coupling with the glycolytic pathway, *β*-oxidation, and the TCA cycle, whereas NADP^+^ to NADPH conversion is accomplished by the pentose phosphate pathway. NADH is used for ATP production in the mitochondria via the electron transport chain, while NADPH, a crucial antioxidant, is used for fatty acid biosynthesis, which provides lipids for membrane biogenesis and confers a survival advantage to cancer cells. As expected, IDF-11774 treatment yielded reduced ATP levels, resulting in an elevated AMP/ATP ratio. The activation of AMPK and suppression of mTOR phosphorylation were also observed in the presence of IDF-11774. It is further likely that IDF-11774 inhibited energy production-related metabolism of cancer cells, because IDF-11774 caused significant glucose-dependent cell death and the suppression of mitochondrial oxidative phosphorylation.

Notably, we revealed that IDF-11774 suppressed the growth of various cancer cells *in vitro* and *in vivo*. In particular, IDF-11774 significantly suppressed tumor growth in both Caki-1 and 786-O renal cancer cells regardless of the presence of a VHL mutation, which is HIF-1*α* independent and differs from the effects of other HIF-1 inhibitors that function only in cancer cells containing wild-type VHL.^9^ Additionally, it was previously reported that combination treatment using the HIF inhibitor BAY 87-2243 and the BRAF inhibitor vemurafenib potentially yielded enhanced tumor growth inhibition in nude mice bearing a BRAF mutant melanoma xenograft.^34^ Similarly, IDF-11774 provided substantial inhibition of tumor growth when combined with sunitinib, sorafenib, or the HER2 inhibitor lapatinib. Therefore, we suggest that IDF-11774 represents a metabolic inhibitor suitable for use in cancer therapy to cure cancers containing mutations in KRAS, PTEN, EGFR T790M, p53, PI3K, and VHL, alone or in combination with other anticancer drugs or radiotherapy.

In summary, we report a novel HIF-1 inhibitor, IDF-11774, which suppressed HIF-1*α* accumulation in colorectal cancer cells *in vitro* and *in vivo*. Additionally, IDF-11774 inhibited glucose uptake, ECAR, and OCR, leading to decreased glucose-dependent energy metabolism. Metabolic analysis of cells treated with IDF-11774 detected significant changes in the levels of glycolysis and TCA cycle metabolites. Furthermore, IDF-11774 exhibited significant efficacy in inhibiting the growth of tumors bearing mutations regulating cancer metabolism. Taken together, our results demonstrate that IDF-11774 targets cancer metabolism to suppress the growth of cancer cells.

## Materials and Methods

### Materials

IDF-11774 were synthesized as described previously.^18^ Sunitinib and 5-FU were obtained from Sigma-Aldrich (St. Louis, MO, USA). Sorafenib and lapatinib were purchased from Santa Cruz Biotechnology (Dallas, TX, USA). Stock solutions of compounds were prepared in DMSO at 10 mM and stored at −20 °C. The following antibodies were used: anti-*β*-actin (sc-47778), anti-ACC*α* (sc-30212), and anti-AMPK*α* (sc-25792) from Santa Cruz Biotechnology; anti-HER2 (2242), anti-mTOR (2983), anti-p-mTOR (2971), anti-pACC (3661), anti-4EBP1 (9644), anti-p-4EBP1 (9459), and anti-p-AMPK (2531) from Cell Signaling Technology (Beverly, MA, USA); anti-HIF-1*α* (610958) and anti-cyclin D1 (554180) from BD Biosciences (San Diego, CA, USA); anti-*β*-tubulin (ab15568) from Abcam (Cambridge, MA, USA).

### Cell culture

The following human cancer cell lines were obtained from the Bioevaluation Center at the Korea Research Institute of Bioscience and Biotechnology (KRIBB): HCT116 colon cancer cells, non-small-cell lung cancer adenocarcinoma A549 and NCI-H1975, pancreatic cancer MIA-PaCa-2, prostate cancer PC-3, and renal cancer Caki-1 and 786-O. Cells were cultured in a 5% CO_2_ atmosphere at 37 °C in Dulbecco’s modified Eagle’s medium (Gibco, Carlsbad, CA, USA) or RPMI (Gibco) supplemented with 10% fetal bovine serum (Gibco), 100 U/ml penicillin, and 100 *μ*g/ml streptomycin (Gibco). Hypoxic conditions were achieved by placing the cells in a 1% O_2_, 94% N_2_, and 5% CO_2_ multigas incubator (Sanyo, Osaka, Japan).

### Western blot analysis

Western blot analysis was performed as described previously.^35^ Cells were lysed with 1 × RIPA buffer (Millipore, Billerica, MA, USA) containing 1 mM Na_3_VO_4_, 1 mM sodium fluoride, 1 mM PMSF, and a protease inhibitor cocktail (Roche, Basel, Switzerland), and the protein concentrations of the resulting lysates were quantified using a BCA Assay Kit (Bio-Rad, Hercules, CA, USA). Western blot signals were detected using an Enhanced Chemiluminescence (ECL) Kit (Millipore).

### Quantitative real-time PCR

Real-time PCR was performed using RT^2^ SYBR Green qPCR Mastermix (Qiagen, Venlo, The Netherlands). Data were analyzed using the Rotor-Gene 6000 Series Software (Corbett Research, Cambridge, UK). VEGF, EPO, GLUT1, PDK1, and HIF-1*α* primers were purchased from Bioneer (Daejeon, Korea).

### *In vivo*/*in vitro* HIF-1 reporter assay

HRE-luciferase activity was measured as described previously.^36^ For bioluminescence imaging assays, IDF-11774 (50 mg/kg) was administered orally to Balb/c nude mice bearing tumors (100 mm^3^) formed from HCT116 cells (1 × 10^7^) expressing HRE-luciferase by subcutaneous injection. After intraperitoneal injection of d-luciferin, bioluminescence images of each tumor were analyzed using the IVIS Lumina II Luminescence Imaging System (Caliper Life Science, Alameda, CA, USA) and Living Image Software (Caliper Life Science).

### *In vitro* tube formation assay

*In vitro* tube formation assays were performed using HUVECs as described previously.^16^ The tubule branches were observed under a microscope and photographed.

### Chicken CAM assay

The CAM assay was performed as described previously.^37^ IDF-11774 (20 *μ*g per egg)- or Sunitinib (10 *μ*g per egg)-loaded thermanox coverslips (Nalge NUNC, Rochester, NY, USA) were laid on the CAM surface.

### Measurement of glucose uptake

Glucose uptake was measured as described previously.^38^ HCT116 cells in Krebs-Ringer phosphate buffer were incubated with 2DG (0.5 *μ*Ci/ml), and tritium activity was measured by Tri-Carb Liquid Scintillation Counting (Perkin-Elmer, Wellesley, MA, USA).

### Measurement of glycolytic flux and mitochondrial respiration

The ECAR was measured using a XF-24 extracellular flux analyzer (Seahorse Biosciences), according to the manufacturer’s instructions. The OCR was measured as described previously.^25^

### ^1^H-NMR analysis of metabolites

Polar metabolites were extracted from cells with solvent composed of methanol, water, and chloroform. ^1^H-NMR spectra were acquired on a Bruker Avance III HD 800 MHz FT-NMR Spectrometer (Bruker BioSpin Co., Billerica, MA, USA) at 298 K using a 5-mm triple-resonance inverse cryoprobe with Z-gradients. The 1D NOESY pulse sequence was applied to suppress the residual water signal. For each sample, 256 transients were collected into 64 000 data points using a spectral width of 16 393.4. All NMR spectra were phased and baseline corrected using Chenomx NMR suite version 7.1 (Chenomx Inc., Edmonton, AB, Canada). Metabolite identities were confirmed based on total correlation spectroscopy (2D ^1^H-^1^H TOCSY) and spiking experiments. Quantification was achieved using the 800 MHz library from Chenomx NMR Suite version 7.1. The amount of each metabolite was calculated according to the number of cells present in the additional sets of sample dishes prepared using the same procedures.

### Xenograft model

All animal experimental protocols were approved by the bioethics committee of the Korea Research Institute of Bioscience and Biotechnology. Cancer cells were injected subcutaneously into 4- to 6-week-old female Balb/c nude mice to generate tumors (5 mice per group). When the tumors grew to 100 mm^3^, IDF-11774 was administered orally (per oral) or intravenously for 15 days. Tumor volumes (*V*) were determined using the following equation: *V* (mm^3^)=(length × width × height) × 0.5. Percentage tumor growth inhibition (%TGI) values were calculated for each treatment group (*T*) versus the control (*C*) using initial (i) and final (f) tumor volumes, according to the formula: %TGI=(1 – [*T*_f_ – *T*_i_]/[*C*_f_ – *C*_i_]) × 100.

### Statistical analysis

Differences between results were analyzed using Student’s *t*-test for unpaired observations and Dunnett’s test for multiple comparisons.

## Figures and Tables

**Table 1:** *In vivo* anticancer efficacy of IDF-11774 in various tumor xenograft models

**Cells**	**Treatment**	**Dose (mg/kg)**	**Route**	**% TGI**
A549	IDF-11774	60	p.o.	51
NCI-H1975	IDF-11774	50	p.o.	32
MIA-PaCa-2	IDF-11774	60	p.o.	48
PC-3	IDF-11774	60	p.o.	62
Caki-1	IDF-11774	60	p.o.	34
786-O	IDF-11774	100	p.o.	62

Abbreviations: p.o., per oral; TGI, tumor growth inhibition
