# Supplementary figures and images for: The novel hypoxia-inducible factor-1α inhibitor IDF-11774 regulates cancer metabolism, thereby suppressing tumor growth
Source: Cell Death Dis. 2017 Jun 1;8(6):e2843–. doi: 10.1038/cddis.2017.235 (PMC5520894; doi:10.1038/cddis.2017.235)

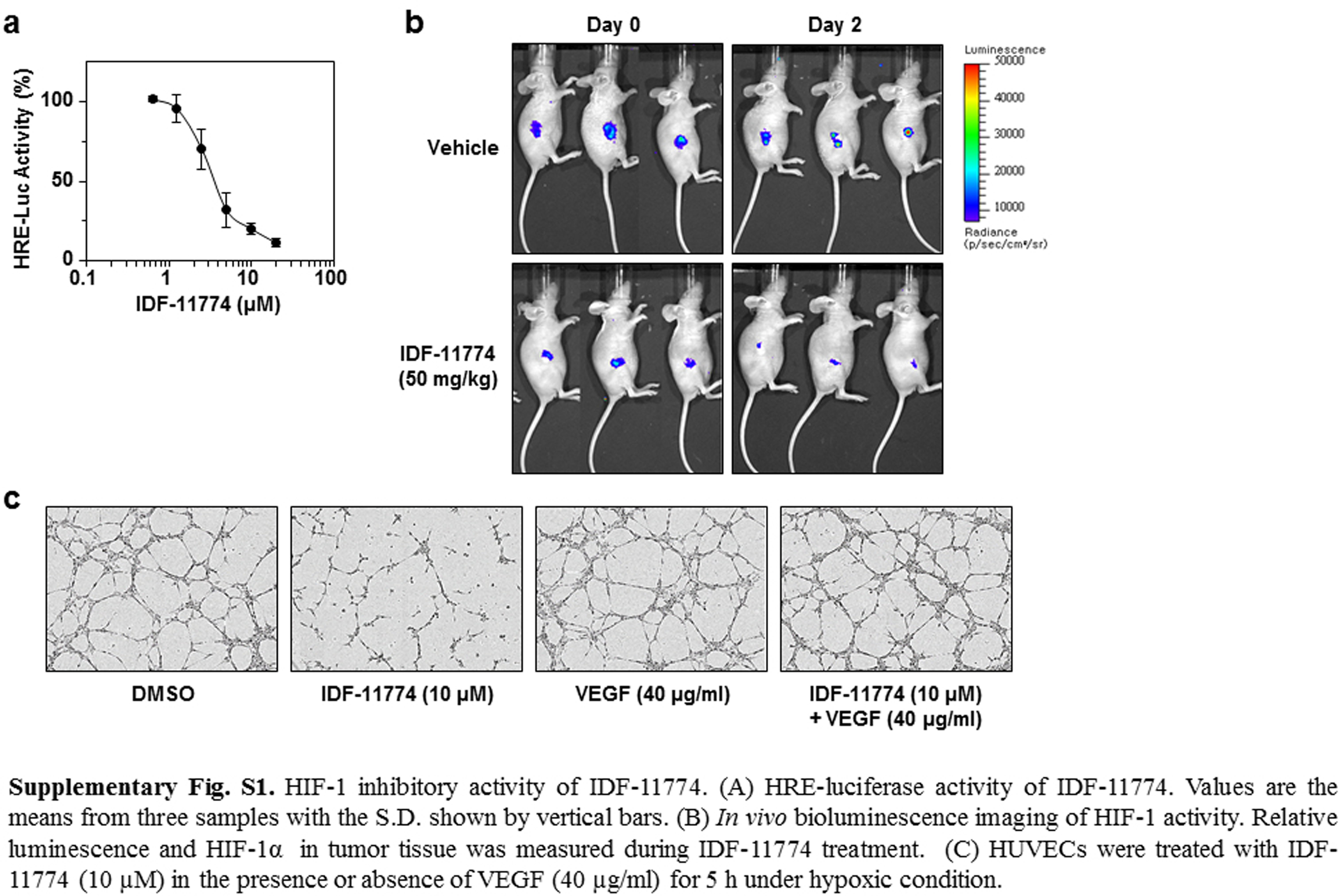

Supplement: Supplementary Figure 1 [file cddis2017235x1.tif]

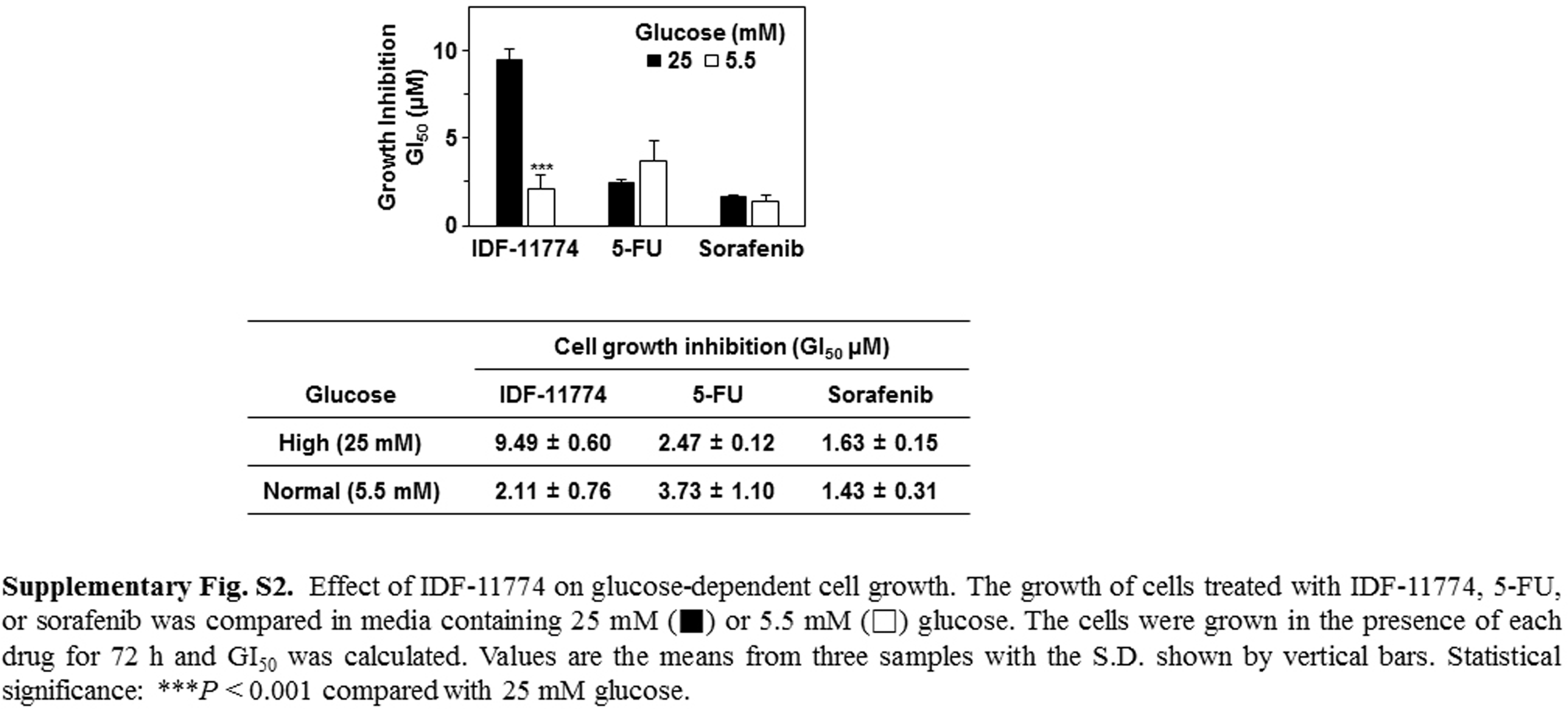

Supplement: Supplementary Figure 2 [file cddis2017235x2.tif]

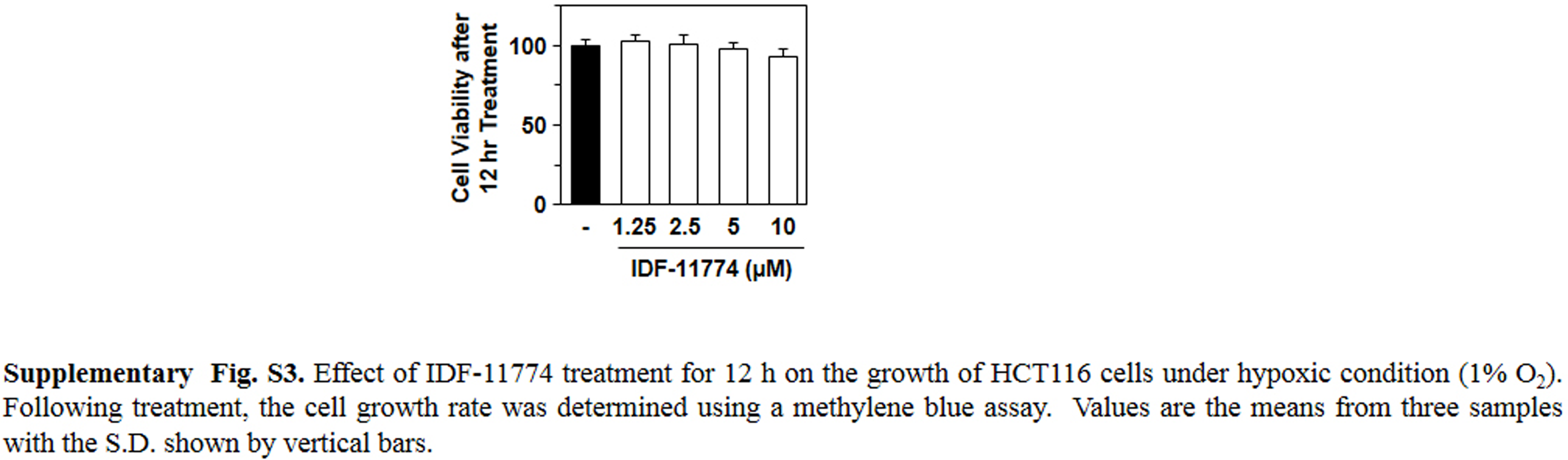

Supplement: Supplementary Figure 3 [file cddis2017235x3.tif]

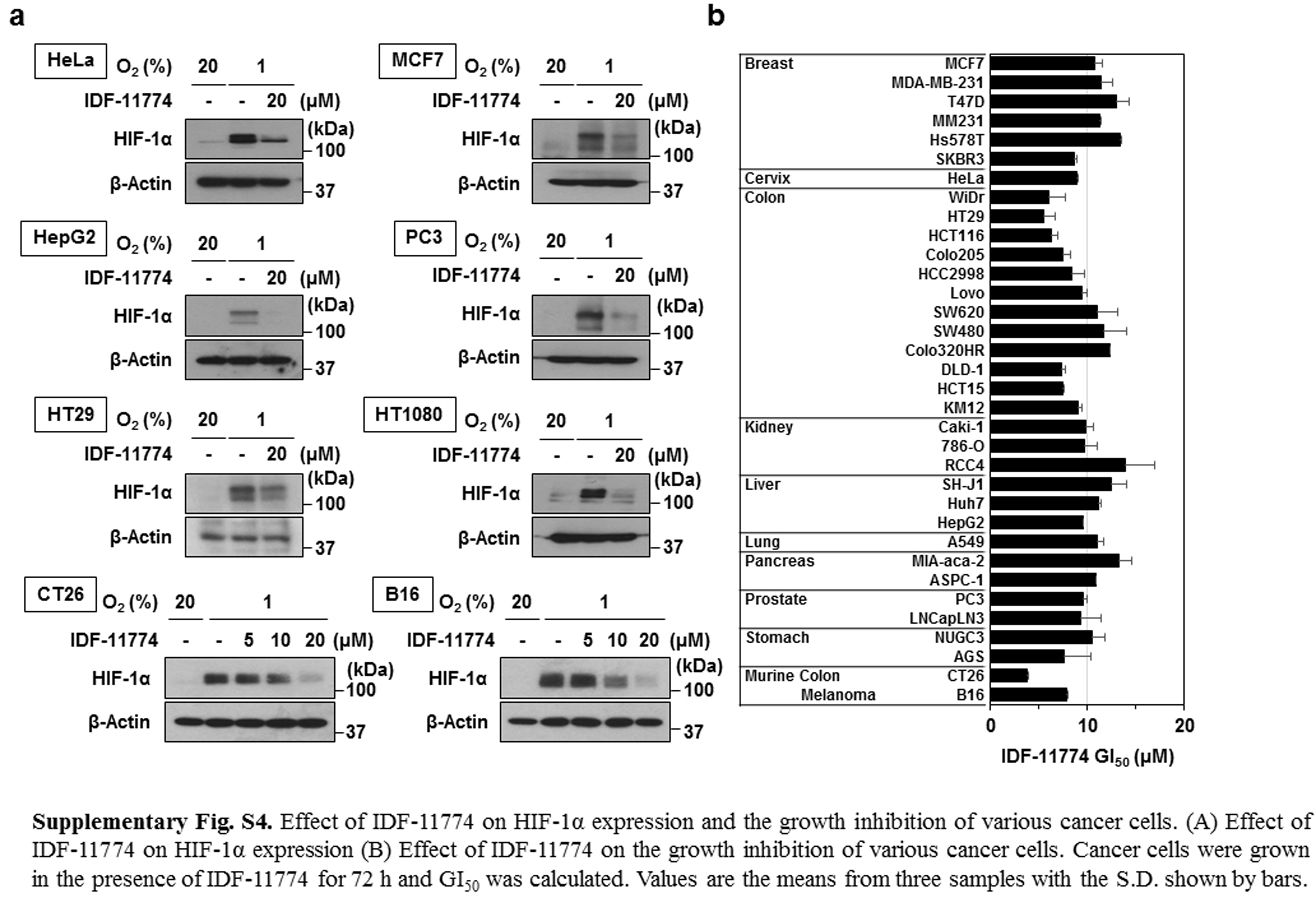

Supplement: Supplementary Figure 4 [file cddis2017235x4.tif]

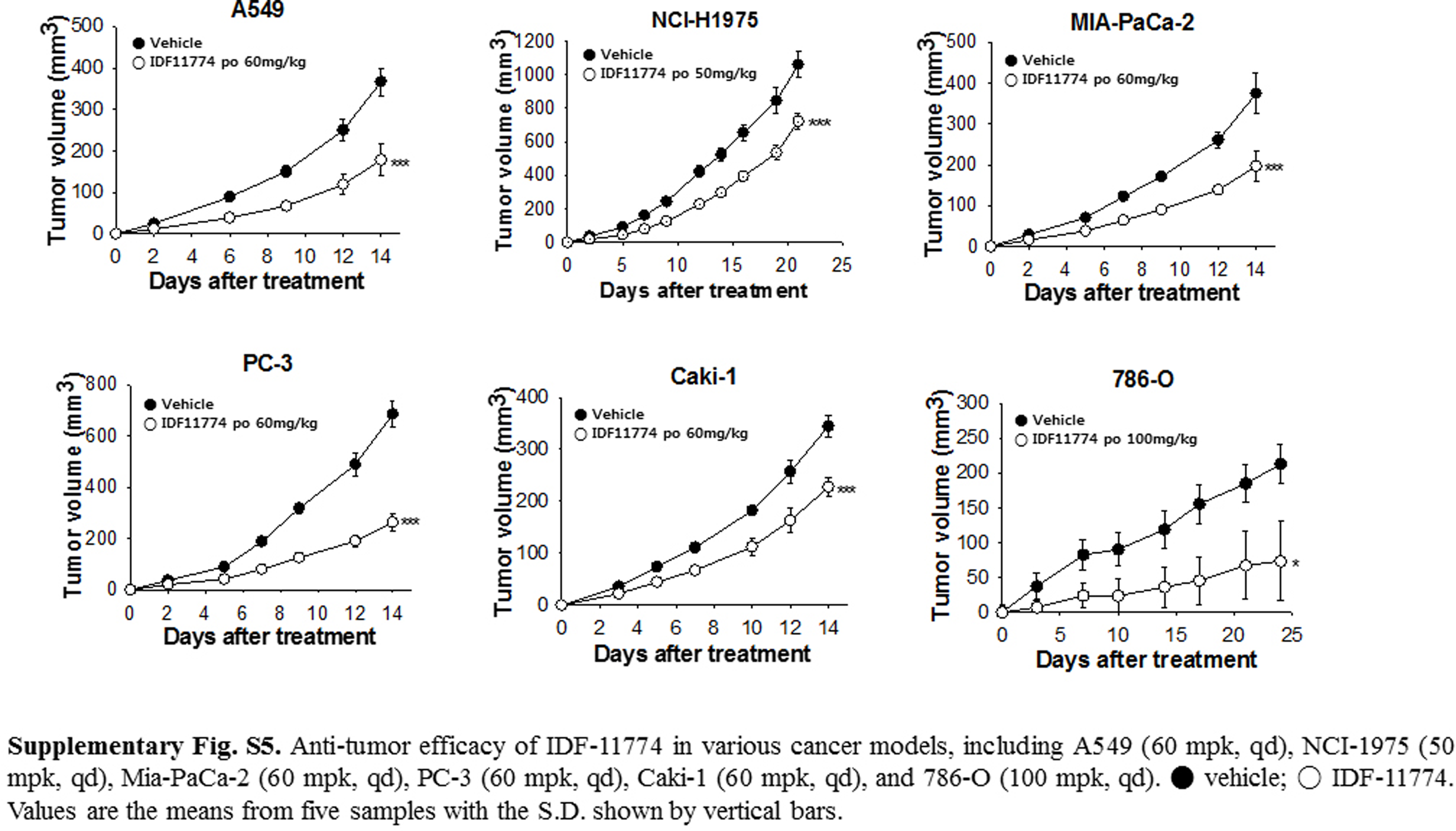

Supplement: Supplementary Figure 5 [file cddis2017235x5.tif]

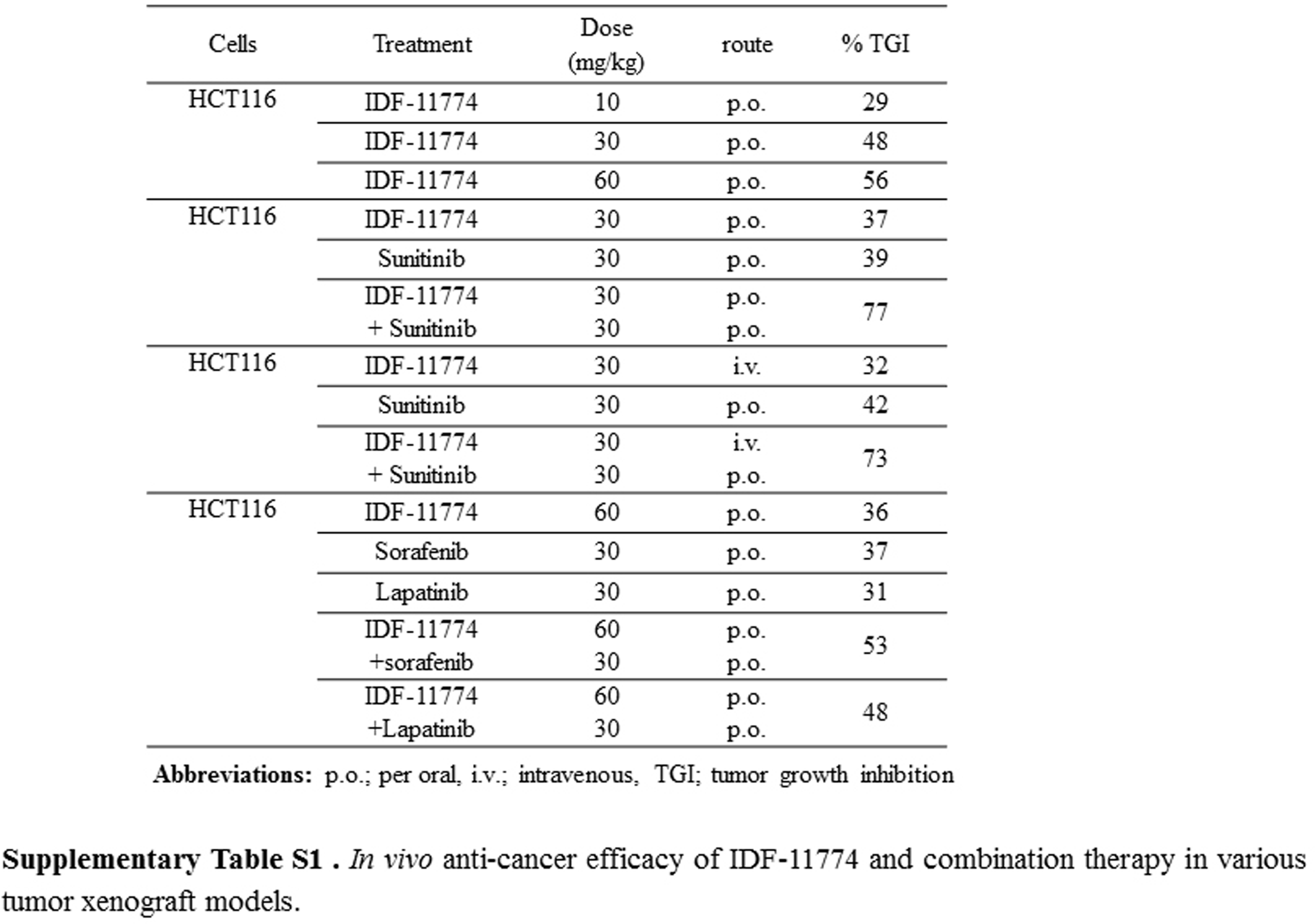

Supplement: Supplementary Table 1 [file cddis2017235x6.tif]
